# PCSK9-mediated degradation of cell-surface LDL receptors impairs human CD8+ T cell effector functions

**DOI:** 10.1016/j.isci.2026.114859

**Published:** 2026-02-07

**Authors:** Angela Markovska, Lara F. Lommers, Alejandra Bodelón, Patrick Greve, Leonie C. van Vark-van der Zee, Monique T. Mulder, Jeanine E. Roeters van Lennep, Noam Zelcer, Henk S. Schipper, Marianne Boes

**Affiliations:** 1 Center for Translational Immunology, University Medical Centre Utrecht, Utrecht, the Netherlands; 2 Department of Biomolecular Health Sciences, Faculty of Veterinary Medicine, Utrecht University, Utrecht, the Netherlands; 3 Department of Internal Medicine Laboratory, Erasmus MC Cardiovascular Institute. Erasmus University Medical Center, Rotterdam, the Netherlands; 4 Department of Medical Biochemistry, Amsterdam UMC Location AMC, University of Amsterdam, Amsterdam, the Netherlands; 5 Amsterdam UMC, Amsterdam Cardiovascular Sciences Institute, Amsterdam, the Netherlands; 6 Amsterdam UMC, Amsterdam Gastroenterology, Endocrinology and Metabolism Institute, Amsterdam, the Netherlands; 7 Department of Pediatric Cardiology, Sophia Children’s Hospital, Erasmus Medical Center, Rotterdam, the Netherlands; 8 Pediatrics Department, University Medical Center, Utrecht, the Netherlands

**Keywords:** Immunology, Cell biology

## Abstract

Proprotein convertase subtilisin/kexin type 9 (PCSK9) regulates circulating cholesterol levels by binding hepatic low-density lipoprotein (LDL) receptors (LDLRs) and directing them to lysosomal degradation. Beyond the liver, PCSK9 expression in multiple cancers, including colorectal, hepatocellular, and head and neck carcinomas, correlates with poor survival. We hypothesized that PCSK9 promotes LDLR degradation on CD8^+^ T cells, limiting cholesterol uptake and impairing antitumor immunity. Treatment of activated human CD8^+^ T cells from healthy donors with recombinant PCSK9 reduced surface LDLR and ICAM-1 expression, granzyme B secretion, and proliferation. The effects of PCSK9 treatment were reversed by PCSK9 inhibition or by culturing cells under lipoprotein-deprived conditions, confirming LDLR dependence. CD8^+^ T cells from patients with homozygous familial hypercholesterolemia, who harbor inactivating *LDLR* mutations, exhibited reduced proliferation and ICAM-1 expression upon activation. Together, these findings identify PCSK9 as a potential therapeutic target to enhance CD8^+^ T cell-mediated antitumor immunity.

## Highlights

• Tumor-infiltrating lymphocytes exhibit dysregulated cholesterol metabolism
• Secreted PCSK9 downregulates cell surface LDL receptors on CD8^+^ T cells
• PCSK9 exposure decreases ICAM-1 expression and granzyme B secretion by CD8^+^ T cells
• CD8^+^ T cells with defective LDL receptor signaling show reduced functional capacity

## Graphical abstract

**Figure:**
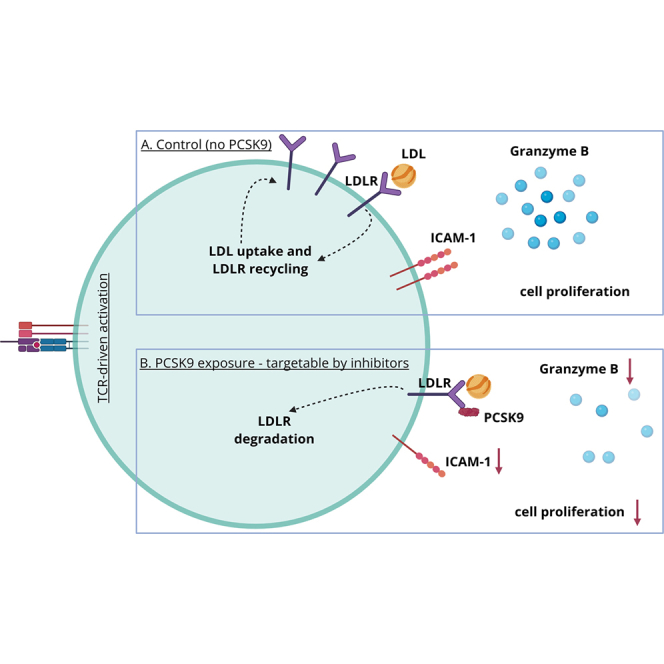

## Introduction

The tumor microenvironment (TME) plays a crucial role in shaping antitumor immune responses. Among the key immune players, CD8^+^ T cells serve as potent effectors capable of recognizing and eliminating malignant cells. However, tumors employ multiple mechanisms to evade immune surveillance, including immune checkpoint engagement, secretion of immunomodulatory factors, and competition for nutrients.^1^ Recent evidence suggests that proprotein convertase subtilisin/kexin type 9 (PCSK9), a serine protease primarily known for its role in cholesterol metabolism, is also secreted by tumor cells and contributes to cancer progression.^2^^,^^3^^,^^4^^,^^5^

The exposure to PCSK9 promotes lysosomal degradation of low-density lipoprotein (LDL) receptor (LDLR) in the liver, thereby reducing LDL clearance and increasing circulating LDL cholesterol levels.^6^ While PCSK9 has been extensively studied in the context of lipid metabolism and cardiovascular disease, its role in the immune landscape of tumors is only emerging. Recent studies, primarily in mouse models, indicate that PCSK9 can impair antitumor immunity, possibly by downregulating the LDLR surface levels on CD8^+^ T cells and thereby hampering their function.^3^^,^^4^^,^^5^^,^^7^ Specifically, studies in LDLR-deficient mice showed impaired CD8^+^ T cell priming, expansion, and T cell receptor (TCR) signaling, all of which are essential for raising effective antitumor responses.^7^^,^^8^ Mechanistically, LDLR was shown to interact with the TCR complexes.^7^ Tumor-derived PCSK9 within the TME can downregulate LDLR levels on mouse CD8^+^ T cells, limiting their effector functions.^7^ Beyond LDLR regulation, PCSK9 has also been implicated in immune evasion mechanisms through its ability to degrade the major histocompatibility complex class I (MHC-I). This reduces antigen presentation on tumor cells, further impairing CD8^+^ T cell recognition and cytotoxicity. Lastly, PCSK9 inhibition enhances the effect of PD-1 immune checkpoint therapy, leading to enhanced CD8^+^ T cell infiltration, improved anti-tumor responses, and significant suppression of tumor growth in mouse models of colorectal and lung cancer.^4^

While experiments done in mouse tumor models highlight PCSK9 as a promising therapeutic target in cancer immunotherapy, its direct effects on human CD8^+^ T cell responses remain incompletely understood. Specifically, it remains unclear whether PCSK9 regulates LDLR expression in human CD8^+^ T cells and which aspects of their immune function are consequently affected. Unraveling these mechanisms in human immune cells could provide critical insights into immune evasion strategies and inform novel therapeutic approaches in cancer treatment.

Here, we investigated the direct impact of secreted PCSK9 on the CD8^+^ T cell response using three experimental human models: (1) recombinant PCSK9 stimulation of primary human CD8^+^ T cells activated polyclonally and (2) in an antigen-specific manner and (3) primary CD8^+^ T cells from patients with homozygous familial hypercholesterolemia (hoFH) and genetically impaired LDLR function. Our findings support that PCSK9-induced LDLR degradation on CD8^+^ T cells impairs their activation and proliferation by reducing CD8^+^ T cell ICAM-1 expression, diminishing their granzyme B-mediated cytotoxicity, and hindering their cell proliferation. Notably, inclusion of the PCSK9 inhibitor alirocumab in the cell cultures reversed these immune inhibitory effects. Our results offer new insights into immune evasion mechanisms and highlight potential strategies to enhance immunotherapy efficacy in PCSK9-expressing tumors.

## Results

### Tumor-infiltrating lymphocytes from advanced colorectal tumors exhibit impaired activation and cholesterol metabolism gene signatures

**Figure 1:**
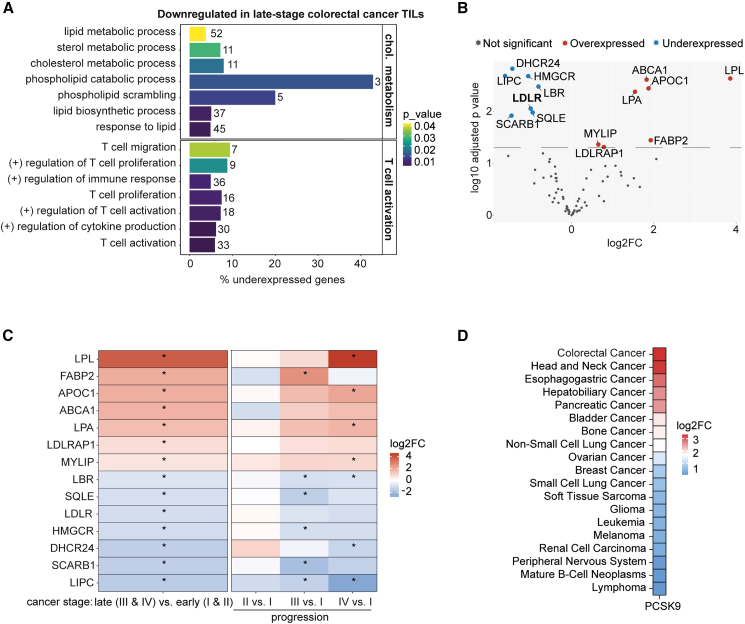
TILs from patients with colorectal cancer show dependency on cholesterol synthesis and uptake (A) Significant enriched Gene Ontology of Biological Process (GO:BP) involved in cholesterol metabolism and T cell activation of downregulated genes in late stage (III and IV) vs. early stage (I and II) TILs from colorectal cancer patients. The number of downregulated genes involved in each GO is indicated beside the bars, and the percentage represents their proportion relative to the total number of genes involved in that function. Total number of patients with colorectal cancer is 18 (stage I, *n* = 5; stage II, *n* = 5; stage III, *n* = 3; stage IV, *n* = 5). The number of genes per pathway is indicated in the graph. (B) Volcano plot of the genes involved in the Cholesterol Metabolism KEGG (hsa04979) and Wiki Pathway (WP5304) representing differential gene expression (log_2_FC) in late-stage vs. early-stage TILs from colorectal cancer patients. Significantly under- and overexpressed genes are highlighted and labeled. (C) Heatmap of significantly (marked with an asterisk) differentially expressed genes from (B), presented as log_2_FC. The left panel compares late- to early-stage colorectal cancer, while the right panel illustrates gene expression changes across disease progression (stage II vs. I, stage III vs. I, and stage IV vs. I). (D) PCSK9 mRNA levels are shown as log_2_fold change (log_2_FC) compared to matched normal tissue. Data were accessed via the cBioPortal for Cancer Genomics (https://www.cbioportal.org/) using the ICGC/TCGA Pan-Cancer Analysis of Whole Genomes dataset.^13^^,^^14^^,^^15^^,^^16^ Data in (A–C) are derived from Saleh et al. (2020).^12^

Impaired infiltration and function of tumor-infiltrating lymphocytes (TILs) are associated with aggressive tumors and therapy resistance. Emerging evidence links cholesterol metabolism to TIL function, particularly in CD8^+^ T cells within the TME.^9^^,^^10^^,^^11^ To investigate the relationship between cholesterol metabolism and TIL function, we analyzed the transcriptomic profiles of TILs from colorectal cancer patients across various disease stages, using data from Saleh et al. (2020).^12^ We compared late-stage (stages III and IV) to early-stage (stages I and II) TILs, performed a functional enrichment analysis, and focused on cholesterol metabolism and T cell activation. In TILs from late-stage colorectal cancer, downregulated genes were enriched for Gene Ontology of Biological Process terms related to cholesterol metabolism and T cell function, suggesting suppression of these pathways during tumor progression (Figure 1A). Specifically, pathways involved in lipid and cholesterol metabolism, and T cell activation, migration, and proliferation, were over-represented among the downregulated genes.

We next analyzed the expression of lipid and cholesterol metabolism genes in TILs from late- vs. early-stage colorectal tumors. Genes encoding proteins involved in increasing cellular cholesterol levels, such as *LDLR*, *HMGCR*, *SCARB1*, and *DHCR24,* were downregulated in TILs from late-stage tumors. Conversely, genes encoding proteins that reduce cellular cholesterol levels, such as *ABCA1* and *MYLIP*, were upregulated in TILs from late-stage tumors (Figure 1B). *MYLIP* encodes the inducible degrader of the LDLR (IDOL), which promotes ubiquitylation of the LDLR and its subsequent lysosomal degradation, resembling the function of secreted PCSK9.^17^^,^^18^ Lastly, a comparison of all disease stages to stage I revealed progressive changes of gene expression in TILs, with increasing changes as cancer advanced (Figures 1C and S1). These findings indicate that cholesterol metabolism is dysregulated in TILs from advanced-stage colorectal cancers.

PCSK9 can bind LDLR, possibly on TILs, promoting its lysosomal degradation and altering cholesterol uptake. To better understand the role of tumor-derived PCSK9, we screened the ICGC/TCGA Pan-Cancer Analysis of Whole Genomes dataset for high PCSK9 expression.^13^^,^^14^^,^^15^^,^^16^ Among the cancers analyzed, colorectal cancer showed the highest upregulation of PCSK9 compared to matched normal tissue (Figure 1D). High PCSK9 expression in cancer patients, including those with colorectal, hepatocellular, and head and neck carcinomas, has been associated with poor survival outcomes.^19^^,^^20^^,^^21^^,^^22^ Conversely, PCSK9 inhibitors, used as cholesterol-lowering agents, are linked to reduced cancer prevalence.^23^^,^^24^ Therefore, we hypothesized that tumor-derived PCSK9 may impair LDLR-mediated lipoprotein uptake in TILs, restricting cholesterol availability and compromising their effector function. This hypothesis provided the biological rationale for our subsequent mechanistic studies in primary human CD8^+^ T cells.

### PCSK9 reduces surface LDLR levels on human CD8^+^ T cells activated *in vitro*

**Figure 2:**
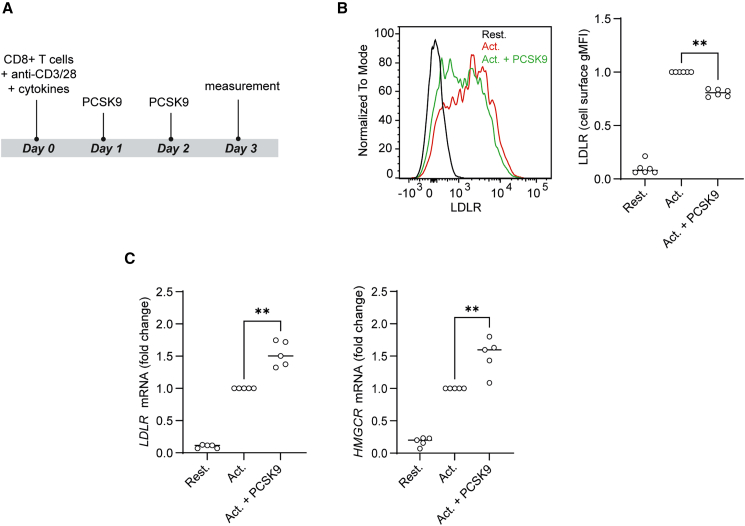
PCSK9 exposure of human *in vitro*-activated CD8^+^ T cells reduces LDLR protein surface expression (A) Schematic representation of the experimental design for *in vitro* CD8^+^ T cell activation in the presence of recombinant PCSK9 (10 μg/mL). (B) Representative cell surface geometric mean fluorescence intensity (gMFI) levels of LDLR on CD8^+^ T cells measured by flow cytometry after 3 days of *in vitro* activation as described in (A). To visualize cell surface LDLR, CD8^+^ T cells were stained with anti-LDLR antibody (clone C7) for 30 min at 4 °C. gMFI levels were normalized to the activated (Act.) control. Two-tailed Mann-Whitney test where ∗∗*p* < 0.01 compared to activated cells, *n* = 6 healthy donors. Each dot represents data from a separate donor, and lines depict medians. (C) Normalized *LDLR* and *HMGCR* mRNA levels measured by qPCR in CD8^+^ T cells measured after 24 h of activation with anti-CD3/28 and cytokines with or without PCSK9 (10 μg/mL) supplementation. Two-tailed Mann-Whitney test where ∗∗*p* < 0.01, *n* = 6 healthy donors. Graphs show independent donors normalized to PCSK9-untreated cells. Each dot represents data from a separate donor, and lines depict medians.

We first examined whether PCSK9 reduces the cell surface levels of LDLR on CD8^+^ T cells. For this, we activated CD8^+^ T cells (anti-CD3/CD28 Dynabeads with cytokines) in the presence of recombinant PCSK9 (10 μg/mL based on prior studies and our dose-response experiments^7^; data not shown) and measured LDLR surface levels by flow cytometry (Figure 2A). LDLR expression was low in resting CD8^+^ T cells but increased upon activation (Figure 2B). CD8^+^ T cells activated in the presence of PCSK9 exhibited lower LDLR cell surface abundance compared to cells activated without PCSK9 (Figure 2B). The effect was modest but statistically significant and consistent across donors.

Next, we assessed whether PCSK9-treated CD8^+^ T cells compensate for reduced LDLR surface levels by upregulating genes involved in lipoprotein uptake and cholesterol biosynthesis. Using the same experimental setup (Figure 2A), we measured mRNA levels of *LDLR* along with *HMGCR*, which encodes the rate-limiting enzyme in the cholesterol biosynthesis pathway. The mRNA levels of *LDLR* and *HMGCR* increased markedly when CD8^+^ T cells were activated in the presence of PCSK9. Taken together, PCSK9 exposure reduces LDLR cell surface occupancy on CD8^+^ T cells. While the cells upregulate LDLR transcripts to compensate, surface levels of LDLR remain reduced.

### PCSK9 reduces the effector functions of human CD8^+^ T cells activated *in vitro*

PCSK9-mediated LDLR degradation in T cells may alter cellular cholesterol content. We, therefore, examined whether PCSK9-induced LDLR reduction affects the formation of cholesterol-rich lipid microdomains in the plasma membrane of CD8^+^ T cells. We assessed lipid microdomains by staining with fluorescein isothiocyanate-conjugated cholera toxin B (CTB), which binds GM1 gangliosides within these domains, and fluorescence was quantified by flow cytometry. Compared with control conditions, PCSK9-treated CD8^+^ T cells showed decreased CTB staining, indicating impaired lipid microdomain formation (Figure S2A).

**Figure 3:**
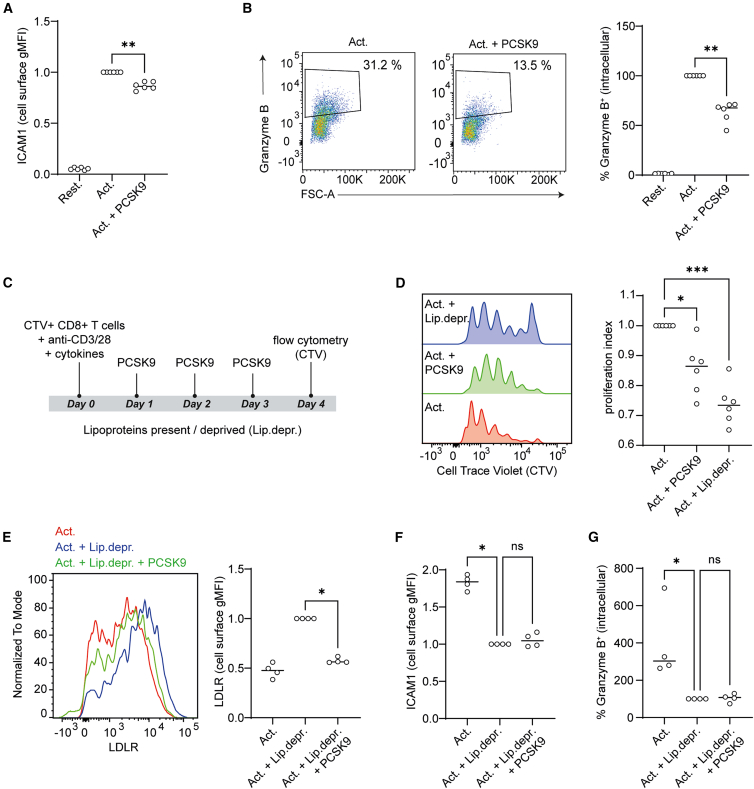
PCSK9 exposure of activated CD8^+^ T cells induces a decrease in ICAM-1 expression and granzyme B production, in anti-CD3/anti-CD28 activation cultures (A) Normalized ICAM-1 cell surface gMFI levels on CD8^+^ T cells measured at day 3 of activation with flow cytometry. Two-tailed Mann-Whitney test where ∗∗*p* < 0.01 compared to PCSK9 untreated, activated cells, *n* = 6 healthy donors. Each dot represents data from a separate donor, and lines depict medians. (B) Intracellular granzyme B levels measured with flow cytometry. Two-tailed Mann-Whitney test where ∗∗*p* < 0.01 compared to PCSK9 untreated, activated cells, *n* = 6 healthy donors. Each dot represents data from a separate donor, and lines depict medians. (C) Schematic representation of the experimental design for *in vitro* CD8^+^ T cell proliferation in the presence of recombinant PCSK9 (10 μg/mL). (D) CTV proliferation assay of CD8^+^ T cells; CTV dilution measured with flow cytometry. Kruskal-Wallis test with Dunn’s multiple comparisons test where ∗*p* < 0.05, ∗∗∗*p* < 0.001 compared to PCSK9 untreated, activated cells, *n* = 6 healthy donors. Each dot represents data from a separate donor, and lines depict medians. (E) LDLR cell surface gMFI levels on CD8^+^ T cells measured with flow cytometry. Two-tailed Mann-Whitney test where ∗*p* < 0.05, *n* = 4 healthy donors. Each dot represents data from a separate donor, and lines depict medians. (F) ICAM-1 cell surface gMFI levels on CD8^+^ T cells measured with flow cytometry. Kruskal-Wallis test with Dunn’s multiple comparisons test where ∗*p* < 0.05, *n* = 4 healthy donors. Each dot represents data from a separate donor, and lines depict medians. (G) Percent of CD8^+^ T cells positive for intracellular granzyme B. Kruskal-Wallis test with Dunn’s multiple comparisons test where ∗*p* < 0.05, *n* = 4 healthy donors. Graphs show independent donors normalized to PCSK9-untreated cells. Each dot represents data from a separate donor, and lines depict medians.

Next, we asked whether PCSK9 treatment impacts the function of CD8^+^ T cells. For this, we first measured cell surface levels of activation markers CD25, CD69, and ICAM-1 in the same setting as in Figure 2A. Treatment with PCSK9 reduced ICAM-1 expression on activated CD8^+^ T cells (Figure 3A), whereas CD25 and CD69 expression remained unchanged, at least at the time point tested (Figure S2B; data for CD69 not shown). ICAM-1 is critical for migration and immunological synapse formation.^25^ Furthermore, we investigated whether PCSK9 treatment affects key cytokines involved in CD8^+^ T cell-mediated cytotoxicity, including interferon (IFN)-γ and tumor necrosis factor alpha (TNF-α), as well as the cytolytic molecule granzyme B.^26^ To this end, we activated CD8^+^ T cells as indicated in Figure 2A and measured the levels of intracellular IFN-γ, TNF-α, and granzyme B using flow cytometry. IFN-γ and TNF-α levels remained unaffected (Figures S2C and S2D), but granzyme B levels were markedly reduced in PCSK9-treated CD8^+^ T cells (Figure 3B).

As cholesterol availability is essential for the expansion of T cells,^27^ we next tested whether PCSK9-mediated decrease of LDLR levels impairs CD8^+^ T cell proliferation. We stained CD8^+^ T cells with CellTrace Violet (CTV), activated them in presence of PCSK9 for 4 days, and assessed proliferation by CTV dilution via flow cytometry (Figure 3C). We found that PCSK9 treatment reduced CD8^+^ T cell proliferation, similarly to when cells were cultured in lipoprotein-deprived condition (Figure 3D).

Thus far, we show that PCSK9 decreases the cell surface LDLR expression on CD8^+^ T cells, which correlates with reduced expression of ICAM-1 and granzyme B. Previous studies have shown that PCSK9 also targets molecules other than LDLR, including MHC-I.^4^ To determine whether the effects of PCSK9 in our model stem from reduced LDLR levels, and consequently decreased LDLR-mediated lipoprotein uptake, we evaluated PCSK9 effects under lipoprotein-free conditions. For this, we activated CD8^+^ T cells under control conditions (Figure 2A), in lipoprotein-deprived culture medium, or in lipoprotein-deprived medium supplemented with PCSK9. In lipoprotein-deprived conditions, cells upregulate LDLR to compensate for low intracellular cholesterol. However, without lipoproteins being present in the medium, LDLR cannot mediate uptake, rendering the increased receptor levels functionally ineffective. We confirmed by flow cytometry that PCSK9 also reduced cell surface LDLR levels on CD8^+^ T cells under lipoprotein-deprived conditions (Figure 3E). Next, we measured surface levels of ICAM-1 and intracellular levels of granzyme B and found that lipoprotein deprivation reduced the levels of both in CD8^+^ T cells. PCSK9 treatment under this condition had no additive effect (Figures 3F and 3G). Therefore, PCSK9 effects on CD8^+^ T cells likely stem from reduced lipoprotein uptake. Taken together, PCSK9 treatment of polyclonally activated CD8^+^ T cells decreases proliferation, ICAM-1 expression, and granzyme B production.

### PCSK9 reduces ICAM-1 expression and granzyme B production by CD8^+^ T cells upon antigen-driven stimulation

**Figure 4:**
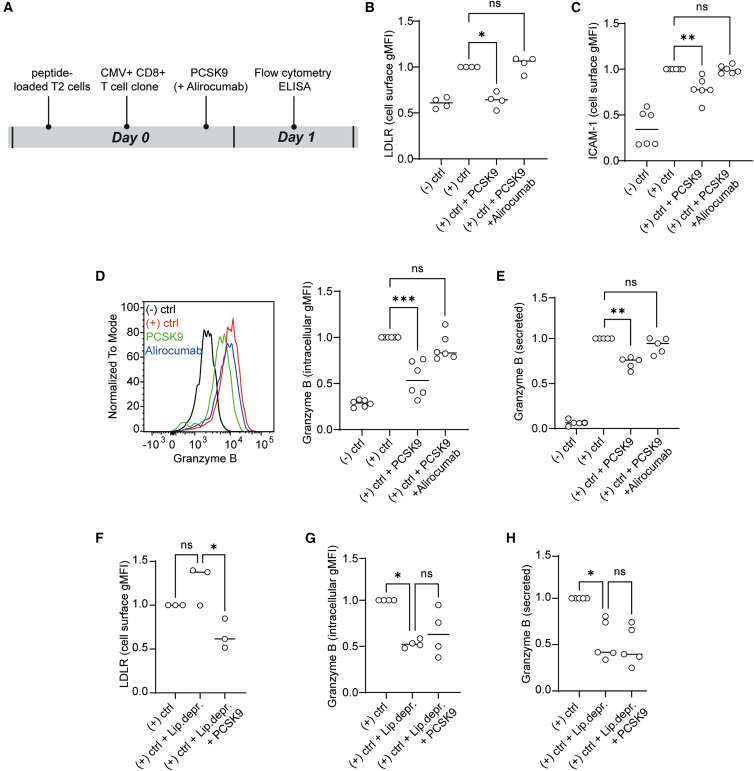
PCSK9 exposure of activated CD8^+^ T cells induces a decrease in ICAM-1 expression and granzyme B production in an antigen-driven activation model (A) Schematic representation of the experimental design, where CD8^+^ T cells specific to NLVPMVATV/HLA-A2 complexes were co-cultured with T2 cells loaded with NLVPMVATV peptide or the irrelevant MART-1-derived ELAGIGILTV peptide. NLVPMVATV peptide is derived from the CMV protein pp65. In the figure, “(−) ctrl” represents the co-culture in presence of the irrelevant MART-1-derived ELAGIGILTV peptide, while in all other conditions, CMV-derived NLVPMVATV peptide was added. Where indicated, recombinant PCSK9 (10 μg/mL) and alirocumab (2 μM) were supplemented to the co-culture. (B) Normalized LDLR cell surface gMFI levels on the CMV-specific CD8^+^ T cells measured with flow cytometry. Kruskal-Wallis test with Dunn’s multiple comparisons test, ∗*p* < 0.05, *n* = 4 independent replicates. Each dot represents an independent replicate, and lines depict medians. (C) Normalized ICAM-1 cell surface expression on the CMV-specific CD8^+^ T cells measured with flow cytometry. 3 h before measuring, cells were treated with GolgiStop (1,500x, BD Biosciences). Kruskal-Wallis test with Dunn’s multiple comparisons test, ∗∗*p* < 0.01, *n* = 6 independent replicates. Each dot represents an independent replicate, and lines depict medians. (D) Normalized intracellular granzyme B levels in the CMV-specific CD8^+^ T cells measured with flow cytometry. 3 h before measuring, cells were treated with GolgiStop (1,500x, BD Biosciences). Kruskal-Wallis test with Dunn’s multiple comparisons test, ∗∗∗*p* < 0.001, *n* = 6 independent replicates. Each dot represents an independent replicate and lines depict medians. (E) Normalized concentrations of secreted granzyme B by CMV-specific CD8^+^ T cells. Kruskal-Wallis test with Dunn’s multiple comparisons test, ∗∗*p* < 0.01, *n* = 5 independent replicates. Each dot represents an independent replicate, and lines depict medians. (F) Normalized LDLR cell surface expression on the CMV-specific CD8^+^ T cells measured with flow cytometry. Kruskal-Wallis test with Dunn’s multiple comparisons test, *n* = 3 independent replicates, ∗*p* < 0.05. Each dot represents an independent replicate, and lines depict medians. (G) Normalized intracellular granzyme B levels in the CMV-specific CD8^+^ T cells measured with flow cytometry. 3 h before measuring, cells were treated with GolgiStop (1,500x, BD Biosciences). Kruskal-Wallis test with Dunn’s multiple comparisons test, ∗*p* < 0.05, *n* = 4 independent replicates. Each dot represents an independent replicate, and lines depict medians. (H) Normalized concentrations of secreted granzyme B by CMV-specific CD8^+^ T cells. Kruskal-Wallis test with Dunn’s multiple comparisons test, ∗*p* < 0.05, *n* = 5 independent replicates. Each dot represents an independent replicate, and lines depict medians.

Thus far, we examined the role of PCSK9 in CD8^+^ T cells activated polyclonally. Previous studies have shown that TILs are recruited to the TME in an antigen-driven manner.^9^ We, therefore, utilized an antigen-driven system with a CD8^+^ T cell clone specific to NLVPMVATV/HLA-A2, a peptide derived from the cytomegalovirus (CMV) protein pp65.^28^^,^^29^ In this setup (Figure 4A), we used T2 cells, which is an antigen-presenting cell line with unstable surface expression of peptide/HLA-A2 complexes due to TAP deficiency.^30^^,^^31^ Adding exogenous peptide stabilized the HLA-A2 molecules, generating cells loaded with NLVPMVATV/HLA-A2 complexes. As a negative control, we used an irrelevant peptide from the melanocyte-derived MART-1 protein (ELAGIGILTV/HLA-A2). To prevent proliferation and metabolic activity, we irradiated the T2 cells before co-culturing them with the antigen-specific CD8^+^ T cell clone. We performed the co-culture with or without PCSK9, and in combination with the PCSK9 inhibitor alirocumab, a clinically used monoclonal antibody that lowers plasma LDL cholesterol levels.^32^^,^^33^ MHC-I levels on T2 cells in our experimental setup were not affected by PCSK9 treatment, possibly because the T2 cells were irradiated and metabolically inactive before co-culture began (Figure S3A).

When the CMV-specific CD8^+^ T cells were co-cultured with T2 cells pulsed with the CMV-derived peptide (positive control), LDLR surface levels were elevated compared to when we used MART-1-derived peptide (negative control; statistical analysis not shown) (Figure 4B). Consistent with our results in Figure 2B, stimulation of the CD8^+^ T cell clone with CMV-derived peptide in the presence of PCSK9 led to reduced LDLR surface levels. This reduction was reversed upon addition of the PCSK9 inhibitor alirocumab (Figure 4B).

We next investigated the effect of PCSK9 on the antigen-driven CD8^+^ T cell response. PCSK9 reduced the surface levels of ICAM-1 (Figure 4C) and the activation marker CD69 on the CMV-specific CD8^+^ T cells stimulated with peptide-pulsed T2 cells (Figure S3B). Furthermore, in this setting PCSK9 also reduced intracellular and secreted granzyme B levels (Figures 4D and 4E). Alirocumab reversed the effects of PCSK9 on ICAM-1, CD69 (Figures 4C and S3B), and granzyme B (Figures 4D and 4E), confirming the specificity and targetability of PCSK9. Treatment with PCSK9 did not alter intracellular IFN-γ levels (Figure S3C).

To test whether the effects of PCSK9 addition depend on LDLR-mediated lipoprotein uptake, we repeated the co-culture under lipoprotein-deprived conditions. Similar to what we observed before, PCSK9 reduced the surface expression of LDLR on the CD8^+^ T cell clone (Figure 4F). The functional effects of PCSK9 on ICAM-1 (Figure 4G) and granzyme B levels (Figure 4H) were abolished when the co-culture was performed under lipoprotein-deprived conditions. Thus, the effects of PCSK9 on ICAM-1 and granzyme B in antigen-driven CD8^+^ T cells are likely linked to reduced LDLR-mediated lipoprotein uptake, supporting our observations obtained by polyclonal T cell stimulation.

### Cultured CD8^+^ T cells from individuals with LDLR gene defects exhibit diminished effector functions

**Figure 5:**
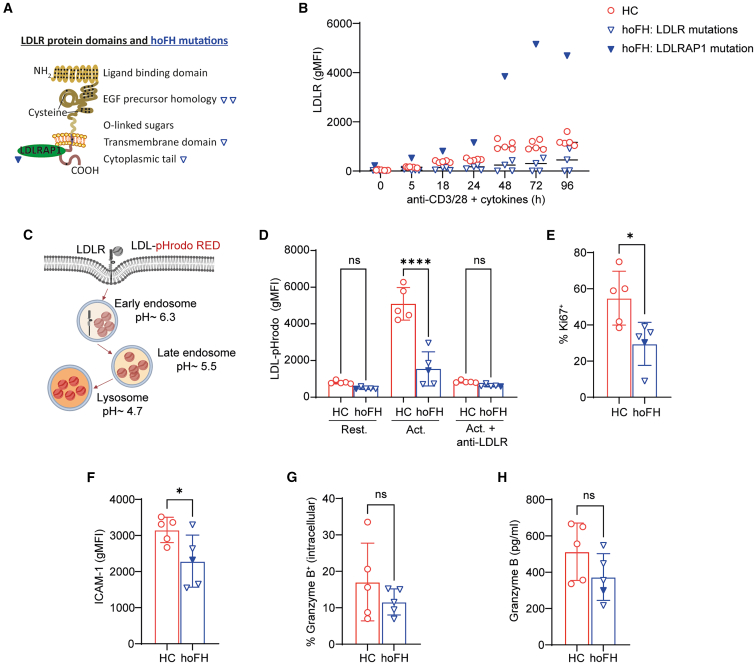
CD8^+^ T cells from individuals with hoFH validate a role of LDLR signaling for CD8^+^ T cell function (A) A schematic overview of the LDLR protein domains and LDLR-adaptor protein 1 (LDLRAP1), highlighting the hoFH mutations included in the experiments with blue reversed triangles. The empty reverse triangle shapes represent hoFH patients with mutations in the LDLR, while the filled reverse triangle shape represents hoFH patients with mutations in the LDLRAP1. (B) Cell surface LDLR gMFI levels on CD8^+^ T cells from hoFH patients (*n* = 5) and healthy controls (HCs) (*n* = 5). Cells were activated for the indicated duration of time with anti-CD3/CD28 Dynabeads and a cytokine mix (interleukin [IL]-2, IL-7, and IL-15). (C) Graphical illustration of the LDL-pHrodo uptake experiment. (D) Uptake of LDL-pHrodo measured by flow cytometry, where CD8^+^ T cells were activated for 24 h, followed by a 2-h culturing in lipoprotein-deprived medium and 2-h incubation with the LDL-pHrodo complex (20 μg/mL). Where indicated, anti-LDLR (5 μg/mL) was added when cells were cultured in lipoprotein-deprived medium. One-way ANOVA with Šídák’s multiple comparisons test, where ∗∗∗∗*p* < 0.0001 (*n* = 5 HCs and *n* = 5 hoFH patients). Each dot represents data from a separate donor, and lines depict medians. (E–H) Cells were activated for 72 h with anti-CD3/CD28 Dynabeads and cytokine mix (IL-2, IL-7, and IL-15). (E) Intracellular Ki67 levels measured with flow cytometry. Two-tailed Mann-Whitney test, where ∗*p* < 0.05 (*n* = 5 HCs and *n* = 5 hoFH patients). Each dot represents data from a separate donor, and lines depict medians. (F) Cell surface ICAM-1 levels measured with flow cytometry. Two-tailed Mann-Whitney test, where ∗*p* < 0.05 (*n* = 5 HCs and *n* = 5 hoFH patients). Each dot represents data from a separate donor, and lines depict medians. (G) Intracellular granzyme B levels measured with flow cytometry. Two-tailed Mann-Whitney test (*n* = 5 HCs and *n* = 5 hoFH patients). (H) Secreted granzyme B levels measured with ELISA. Two-tailed Mann-Whitney test (*n* = 5 HCs and *n* = 5 hoFH patients). Each dot represents data from a separate donor, and lines depict medians.

Our data demonstrated that PCSK9 exposure impairs CD8^+^ T cell function by reducing LDLR surface levels on the cells. We hypothesized that CD8^+^ T cells with inherently defective LDLR-mediated cholesterol uptake would, therefore, also show impaired activation. To test this, we analyzed CD8^+^ T cells from individuals with hoFH (OMIM 143890 and OMIM 603813) carrying bi-allelic pathogenic mutations that lead to dysfunctional LDLR (Figures 5A and S4A).

First, we confirmed that, compared to healthy controls, activated CD8^+^ T cells from individuals carrying *LDLR* mutations have reduced LDLR surface expression (Figure 5B). CD8^+^ T cells from an individual with a mutation in the *LDLR adaptor protein 1* (*LDLRAP1*) had increased LDLR surface expression (Figure 5B), which was expected as mutations in *LDLRAP1* disrupt LDLR internalization, while maintaining normal or elevated surface occupancy of structurally intact LDLR.^34^^,^^35^ To investigate the uptake of LDL cholesterol by the CD8^+^ T cells from hoFH patients, we performed an LDL-pHrodo uptake experiment. For this, we conjugated LDL particles with pHrodo dye, which exhibits fluorescence as the pH levels lower. We activated CD8^+^ T cells with anti-CD3/CD28 Dynabeads for 24 h, followed by 2-h lipoprotein deprivation, after which we added the pHrodo-labeled LDL (20 μg/mL) to the cells for an additional 2 h. As internalized LDL-pHrodo intersects with acidic vesicles of the endolysosomal pathway, it becomes fluorescent, allowing quantification of uptake (Figure 5C). We measured the fluorescent signal by flow cytometry, and as a negative control also included cells pre-treated with an LDLR-blocking antibody. Activated CD8^+^ T cells from hoFH patients showed reduced LDL uptake compared to controls (Figure 5D). Addition of LDLR-blocking antibody reduced LDL uptake in control cells to the levels observed in the hoFH patients (Figure 5D). Our data confirm that activated CD8^+^ T cells from patients with hoFH have impaired LDLR-mediated LDL uptake.

Next, we activated CD8^+^ T cells from hoFH patients and healthy individuals using the same setup as in Figure 2A, but omitting PCSK9 since hoFH patients inherently exhibit reduced LDLR function. As indication of cellular proliferative capacity, we measured intracellular Ki67 expression. CD8^+^ T cells from individuals with hoFH showed reduced levels of Ki67 compared to cells from healthy controls (Figure 5E). Surface CD25 levels were comparable between hoFH and controls (Figure S4B), whereas ICAM-1 levels were reduced on activated CD8^+^ T cells from hoFH patients (Figure 5F). Last, we analyzed intracellular and secreted levels of IFN-γ and granzyme B and observed no significant differences between hoFH patients and healthy controls (Figures S4C, S4D, 5G, and 5H). Granzyme B expression remained preserved in hoFH CD8^+^ T cells, unlike the PCSK9-driven reduction seen in Figures 3 and 4. Taken together, these findings further support that impaired LDLR activity, due to genetic deficiency or through PCSK9-driven degradation, compromises CD8^+^ T cell function.

## Discussion

Cholesterol availability varies not only between tissues but also among different cell types, including those within the TME. Tumor cells and immunosuppressive myeloid cells often accumulate cholesterol, whereas TILs can exhibit a shortage of cholesterol.^27^ This depletion can arise from metabolic reprogramming or competition for limited cholesterol within the TME. For example, a previous study used an adoptive transfer model to introduce antigen-specific CD8^+^ T cells into either vaccinated or tumor-bearing mice. Despite receiving equivalent antigen stimulation, the CD8^+^ T cells isolated from tumors exhibited significantly lower cholesterol levels than those from vaccinated mice, indicating that the TME actively drives cholesterol depletion in TILs.^27^ Inside of T cells, cholesterol contributes to TCR clustering and signaling, through the formation of cholesterol-rich membrane microdomains necessary for effective immune synapse assembly. However, excessive cholesterol can disrupt this process and has also been reported to inhibit receptor clustering, underscoring the importance of a tightly regulated balance.^36^^,^^37^ In the TME, cholesterol accumulation can also drive T cell exhaustion and reduced T cell effector function.^36^ Our findings add to this complexity by showing that PCSK9-dependent disruption of cholesterol homeostasis impairs CD8^+^ T cell function.^6^ We demonstrate across three human models that PCSK9 reduces LDLR surface expression on CD8^+^ T cells and subsequently diminishes their activation, proliferation, and cytolytic capacity. Our findings complement existing studies based primarily on murine models, which demonstrate a correlation between PCSK9 expression, tumor progression, and impaired immune function.^4^^,^^7^^,^^20^

We found that TILs from late-stage colorectal cancer patients exhibit a transcriptional program consistent with diminished activation and reduced cholesterol uptake, synthesis, and metabolic processing. Notably, many downregulated cholesterol metabolism genes are direct targets of sterol regulatory element-binding protein 2 (SREBP2), suggesting impaired SREBP2 activity alongside reduced T cell activation in these tumors.^37^ These findings highlight a tight link between intracellular cholesterol homeostasis and T cell effector function, in line with prior work in human systems and mouse models.^36^^,^^38^^,^^39^^,^^40^^,^^41^ T cell activation itself is known to trigger SREBP2 activation, where mTORC1 signaling during T cell priming promotes SREBP2 processing, possibly by lowering endoplasmic reticulum cholesterol levels.^42^^,^^43^ The association between cholesterol metabolism and T cell activation is not specific to colorectal cancer.^4^^,^^19^^,^^20^ Elevated PCSK9 expression has been observed across multiple cancer types and is associated with poor prognosis, although prior work has relied primarily on transcriptomic data.^44^

Mechanisms that describe how cholesterol contributes to lipid microdomains and receptor clustering are described.^7^^,^^45^ Here, we instead examine the downstream effects of PCSK9-driven LDLR modulation on CD8^+^ T cell function. Our findings underscore the importance of LDLR-mediated lipoprotein uptake in sustaining cytotoxic T cell effector programs. One key mechanism may involve cholesterol-dependent formation and function of lysosomal secretory vesicles that store cytolytic molecules such as granzyme B. Vesicle biogenesis, trafficking, and fusion rely on membrane lipid composition, including cholesterol, which shapes membrane curvature, fluidity, and domain organization necessary for vesicle budding, stability, and docking at the immunological synapse.^46^ Disruption of cholesterol homeostasis, for example, through decreased LDLR expression, likely diminishes intracellular cholesterol availability and impairs lysosomal membrane formation and function. The reduced intracellular cholesterol may lead to defective granzyme B packaging, retention, or release we observed in cultures treated with PCSK9. In contrast to granzyme B, IFN-γ or TNF-α levels were unaffected, aligning with the fact that these cytokines are primarily transcriptionally regulated, whereas granzyme B depends more strongly on vesicular trafficking and cellular metabolic state.^47^ Future studies incorporating metabolic tracing of cholesterol in secretory vesicles and ultrastructural analyses will be important to clarify the underlying mechanisms linking granzyme B secretion and intracellular cholesterol levels in CD8^+^ T cells.

We also observed reduced ICAM-1 surface expression following PCSK9 treatment, consistent with the requirement of ICAM-1 for cholesterol-rich lipid microdomains for stable membrane localization.^48^ Supporting this, PCSK9 inhibition enhanced CD8^+^ T cell migration *in vivo*,^20^ aligning with our observation that PCSK9 reduces ICAM-1 and may thereby influence T cell motility. Prior studies examining PCSK9-mediated ICAM-1 regulation were conducted in endothelial cells,^49^^,^^50^ where ICAM-1 biology differs from T cells. Endothelial work primarily assessed soluble or transcript-level ICAM-1, whereas on T cells, ICAM-1 functions as both an adhesion molecule and an activation marker, and surface protein levels are more relevant for immune function. These context-specific differences likely explain apparent discrepancies across studies. PCSK9 did not significantly alter CD25 expression despite reducing CD8^+^ T cell proliferation. CD25 marks early interleukin-2-dependent activation, whereas proliferation requires sustained metabolic support. Of note, we observed an effect due to PCSK9 treatment on activation markers when we activated cells in an antigen-specific manner for 24 h. A limitation is, therefore, that we may have missed a temporal window of CD25 downregulation. Nonetheless, our findings highlight that cholesterol availability differentially regulates discrete aspects of T cell biology, including adhesion, migration, and proliferative capacity.

In addition to examining the effects of PCSK9-mediated LDLR degradation, we also analyzed CD8^+^ T cells from patients with hoFH, who carry genetic defects in LDLR. These “experiments of nature” offer valuable insight into the physiological relevance of LDLR-dependent lipoprotein uptake in human T cells. Consistent with prior observations in heterozygous FH patients,^8^ we observed impaired proliferative capacity in hoFH CD8^+^ T cells following activation. In this study we assessed Ki-67 expression at day 3 only, and future work with longitudinal time points will be required to better characterize the proliferative dynamics. We also detected reduced ICAM-1 upregulation in hoFH CD8^+^ T cells upon stimulation, mirroring the effects of PCSK9 treatment. In contrast to the work by Bonacina et al.^8^ and our PCSK9-treated healthy donor experiments, we did not detect reduced granzyme B expression in hoFH CD8^+^ T cells. Methodological differences may explain this discrepancy: Bonacina et al. used antigen-specific stimulation and longer cultures, whereas our assays with hoFH cells involved shorter durations and polyclonal activation. In addition, hoFH T cells are chronically exposed to elevated PCSK9 and dysregulated lipid levels *in vivo*, which may trigger compensatory mechanisms that preserve certain effector functions, such as granzyme B production, despite impaired activation and proliferation.

The immunological defects that we observed *ex vivo* were unexpected, as hoFH patients do not report increased cancer incidences. Several factors could contribute to this apparent discrepancy. First, LDLR deficiency may be partially compensated *in vivo* in ways not captured in short-term cultures. Second, the extreme rarity and premature cardiovascular mortality of hoFH patients complicate long-term oncologic surveillance.^51^^,^^52^ Lifestyle modifications common among FH patients, such as healthier diet, increased physical activity, and lower smoking and alcohol use, may further mitigate malignancy risk. More broadly, while systemic hypercholesterolemia does not necessarily increase cancer risk, even reducing risk in some cases in FH patients,^53^ dysregulated cholesterol metabolism within the TME can promote tumor growth and impair antitumor immunity.^54^ Thus, the relationship between cholesterol and cancer is context-dependent: cholesterol is essential for immune cell activation, yet can support tumor progression when locally accumulated or aberrantly regulated.

Taken together, our study underscores a critical role for LDLR-mediated lipoprotein uptake in sustaining CD8^+^ T cell function. We demonstrate that PCSK9-mediated downregulation of LDLR impairs CD8^+^ T cell proliferation and cytolytic capacity through LDLR suppression. These findings in human cells deepen our understanding of the crosstalk between metabolic and immune pathways in the TME and suggest adjuvant therapy based on the targeting of PCSK9 as a promising avenue toward improved efficacy of existing cellular immunotherapies.

### Limitations of the study

A key limitation of our study is that functional experiments were performed using CD8^+^ T cells from healthy donors rather than patient-derived TILs. Further studies might dissect the complex interplay between cholesterol metabolism, TIL function, and tumor progression in clinically relevant models, to identify the specific cellular sources of PCSK9 within tumors and clarify its impact on LDLR expression in TILs. Here, studies using murine cancer models we believe will provide valuable information. From our current study, we also cannot fully exclude LDLR-independent effects of PCSK9 on CD8^+^ T cells, although our data indicate that lipoprotein availability is required for PCSK9-mediated effects. We used wild-type recombinant PCSK9, and we observed approximately 30% reduction of LDLR levels on CD8+ T cells. Future studies may consider using PCSK9 with D374Y gain-of-function mutation to potentially observed more pronounced LDLR decrease as well as functional effects on the CD8^+^ T cells. Last, due to the limited sample size and the exploratory nature of the study, we were not sufficiently powered to assess the influence of sex or gender on the observed outcomes.

## Resource availability

### Lead contact

Requests for further information and resources should be directed to and will be fulfilled by the lead contact, Marianne Boes (m.l.boes@umcutrecht.nl).

### Materials availability

This study did not generate new unique reagents.

### Data and code availability

• This paper analyzes existing, publicly available data, published at https://doi.org/10.1136/jitc-2020-001294.
• This paper does not report original code.
• Any additional information required to reanalyze the data reported in this paper is available from the lead contact upon request.

## Acknowledgments

We thank the members of Boes lab for the thoughtful discussions.

## Author contributions

A.M., H.S.S., and M.B. initiated the study and wrote the manuscript. A.M., L.F.L., and P.G. performed experiments and data analysis. A.B. performed the bioinformatic analysis. All authors contributed to substantial discussion of content and reviewed and approved the final manuscript.

## Declaration of interests

M.B. received funding from Actuate Therapeutics. H.S.S. was partly supported by a Veni grant from the Netherlands Organization for Scientific Research (NWO; 91618150) and is supported by an Erasmus MC Research Fellowship and Starting Grant. N.Z. is supported by a Vici grant from the Netherlands Organization for Scientific Research (NWO; 016.176.643) and an NWO ENW grant (M.22.034; GENESIS). The department of J.E.R.v.L. received finances for an investigator-initiated grant about shared decision making for the choice of PCSK9 inhibitors (Novartis).

## STAR★Methods

### Key resources table

**Table:** 

REAGENT or RESOURCE	SOURCE	IDENTIFIER
**Antibodies**		

LDLR (C7)-PE	Bio Connect	Cat # 10231-R301-P-100; RRID: AB_2860124
IFNy (4S.B3)	BD	Cat# 557844; RRID: AB_396894
Granzyme B (GB11)-FITC	BD	Cat # 560211; RRID: AB_1645488
CD25 (B1.49.9)-AF700	Beckman Coulter	Cat# B92454
ICAM-1 (HCD54)-PB	Biolegend	Cat# 322716; RRID: AB_893384
CD69 (FN50)-PE-Cy7	Biolegend	Cat# 557745; RRID: AB_396851
TNFα (MAB11)-APC	Biolegend	Cat# 502912; RRID: AB_315264
MHC-I (G46-2.6)-PE-Cy7	BD	Cat# 561349; RRID: AB_10612559

**Biological samples**		

PBMCs from healthy donors	Mini Donor Dienst UMCU	N/A
PBMCs from FH patients	Erasmus MC	MEC-2023-0742

**Chemicals, peptides, and recombinant proteins**		

Ficoll-Paque	UMC intern / VWR	3002655
Human Recombinant IL-2	BioLegend	571202
Human Recombinant IL-7	Tebu-Bio	200-07-B
Human Recombinant IL-15	Immunotools	11340153
Fixable Viability Dye eFluor 780	eBioscience	65-0865-14
CellTrace™ Violet Cell Proliferation Kit, for flow cytometry	ThermoFisher Scientific	C34557
NLVPMVATV	Fisher Scientific	15312156
ELAGIGILTV	Fisher Scientific	6-7007-901
beta-2 microglobulin	Bio-Rad	6240-0824
GolgiStop	BD bioscience	554724
KBr	Merck	P0838-250G
pHrodo iFL Green STP Ester (amine-reactive)	Thermo Fisher Scientific	15700847
SYBR Select Master Mix	Bio-Rad	13256519
Ionomycin calcium salt	Sigma	I0634-1MG
phorbol 12-myristate 13-acetate	Sigma	P8139-1mg
CHOLERA TOXIN B SUBUNIT FITC LABELED	Merck	C1655-.5MG

**Critical commercial assays**		

CD8 T cell isolation kit	Miltenyi	130-096-495
T cell activator CD3/CD28 Dynabeads	Gibco	11131D
granzyme B ELISA kit	R&D systems	DY2906-05
IFNγ ELISA kit	Biolegend	430104
RNeasy mini kit	Qiagen	74104
cDNA Synthesis Kit	Bio-rad	1708890
eBioscience™ Foxp3 / Transcription Factor Staining Buffer Set	eBioscience	00-5523-00

**Software and algorithms**		

FlowJo v10	https://www.flowjo.com	N/A
GraphPad Prism v10	https://www.graphpad.com	N/A
gProfiler2	R package	v0.2.3
BD FACSDiva™	https://www.bdbiosciences.com/en-no/products/software/instrument-software/bd-facsdiva-software	N/A

**Deposited data**		

RNA-seq data of tumor-infiltrating CD8^+^ T cells	Saleh R. et al., J Immunother Cancer 2020; supplementary table	https://doi.org/10.1136/jitc-2020-001294

### Experimental model and study participant details

#### Human blood samples

Peripheral blood was collected from male and female healthy volunteers (Mini Donor Dienst, University Medical Center Utrecht) and five patients with homozygous familial hypercholesterolemia (hoFH) using BD Vacutainer tubes containing sodium heparin. All participants provided written informed consent prior to sample collection. The study was conducted in accordance with the Declaration of Helsinki. Ethical approval for the collection of blood from healthy volunteers was obtained from the institutional ethical review board of the University Medical Center Utrecht (protocol number 07-125/C). Healthy control blood samples were obtained from adult donors aged 18-55 years; donors could be of any sex, gender, ancestry, race, or ethnicity. Healthy donors were not allocated to experimental groups based on biological characteristics, and detailed donor demographics were not recorded. Ethical approval for inclusion of five hoFH patients was obtained from the Medical Research Ethics Committee of the Erasmus Medical Center Rotterdam (protocol number MEC-2023-0742). Patient samples were obtained from five individuals (two females and three males), with ages ranging from 28 to 55 years.

#### Cell lines

The T2 cell line (174 × CEM.T2, ATCC CRL-1992; kindly provided by Dr. Sonja Buschow; Erasmus Medical Center, Rotterdam, NL) was used in this study. Cell line identity was verified by the supplier and confirmed by phenotypic characteristics described for T2 cells, including stable MHC class I expression deficiency. The cell line was routinely tested for mycoplasma contamination.

### Method details

#### CD8^+^ T cell purification and culture

Peripheral blood mononuclear cells (PBMCs) were isolated with Ficoll-Paque density gradient centrifugation. CD8^+^ T cells were isolated from full PBMCs using the CD8 T cell isolation kit (Miltenyi) with autoMACS (Miltenyi) automated cell isolation following the manufacturer’s protocol. CD8^+^ T cells were cultured in RPMI 1640 (ThermoFisher Scientific) supplemented with 10% heat-inactivated human serum, 1% penicillin-streptomycin and 2 μM L-glutamine. Where indicated, CD8^+^ T cells were activated with human T cell activator CD3/CD28 Dynabeads (Gibco) in a ratio of beads to CD8^+^ T cells 1:3, and in presence of a cytokine mix consisting of recombinant human IL-2 (25 IU/ml), IL-7 (Tebu-Bio, 20 ng/ml) and IL-15 (Immunotools, 20 ng/ml). Where indicated, recombinant PCSK9, kindly provided by the lab of prof. Noam Zelcer (AMC Amsterdam), was added in concentration 10 μg/ml.

#### CD8^+^ T cell – T2 cell line co-culture

A human CD8^+^ T cell clone specific for a CMV-derived peptide NLVPMVATV was expanded *in vitro* and maintained in RPMI 1640 (ThermoFisher Scientific) supplemented with 10% heat-inactivated human serum, 1% penicillin-streptomycin, 2 μM L-glutamine and IL-2 (50 IU/mL). T2 cells (TAP-deficient, HLA-A2+ cell line) were pulsed with the NLVPMVATV or ELAGIGILTV peptide (both 10 μM) in presence of 5 μg/ml beta-2 microglobulin for 2 hours at 37 °C, washed and irradiated at 25 Gy. T2 cells were co-cultured with the CD8^+^ T cells at a 2:1 ratio. Co-cultures were incubated for 24 hours. Supernatants were collected for cytokine analysis, and T cell activation was assessed via flow cytometry. For intracellular cytokine measurement, cells were incubated with GolgiStop (BD bioscience stock concentration 1500x diluted) for 3.5 hours.

#### Detection of secreted cytokines

CD8^+^ T cell culture supernatants were collected and stored at -80 °C until analyzed. Cytokine levels were measured using specific ELISA kits for granzyme B (R&D systems) and IFNγ (BioLegend) following manufacturer’s protocol. Calorimetric measurements were performed using CLARIOstar ELISA plate reader (BMG Labtech).

#### Lipoprotein isolation and fluorescence labing

Lipoproteins were isolated from human serum (Sanquin) using a previously established ultracentrifugation protocol with KBr gradient (105,000 g for 20 hours with breaks off).^55^ Isolated lipoproteins were fluorescently labeled with pHrodo iFL Green STP Ester (amine-reactive) (Thermo Fisher Scientific), according to supplier instructions. For LDL-pHrodo uptake experiments, cells were incubated with LDL-pHrodo complexes (20 μg/ml) for 2 hours.

#### Quantitative real-time PCR (qRT-PCR)

Total RNA was isolated from the cells using the RNeasy mini kit (Qiagen) according to the manufacturer's protocol. Equal amount of RNA was transcribed into cDNA with the cDNA Synthesis Kit (Bio-Rad). SYBR green qPCR master mix (Bio-Rad) was used to perform the qRT-PCR reaction. The primers used are shown in Table S1. Measurements were done with QuantStudio 3 (Fisher Scientific) and gene expression levels were determined according to the ΔCt methods (relative abundance = 2(-ΔCt)). RPL13A was used as housekeeping gene.

#### Flow cytometry

For intracellular cytokine staining, the cells were additionally stimulated with phorbol 12-myristate 13-acetate (PMA) (20 ng/ml, Sigma) and Ionomycin calcium salt (1 μg/ml, Sigma) for 30 minutes, after which GolgiStop (BD bioscience stock concentration 1500x diluted) was added and further incubated for 3.5 more hours. After the end of the culture experiment, cells were washed with phosphate buffered saline (PBS) and stained with Fixable Viability dye eFluor 780 (eBioscience) for 20 minutes at 4 °C to identify and exclude the dead cells from the analysis. The cells were then washed with FACS buffer (PBS supplemented with 2% FCS and 0.1% sodium azide) followed by a blocking incubation with 10% mouse serum in FACS buffer. The cells were then stained with surface antibodies for 20 minutes at 4 °C. When staining with anti-LDLR antibody, the cells were stained for 30 minutes at 4 °C under gentle agitation. For intracellular staining the cells were fixed and permeabilized using Fixation and Permeabilization buffer (eBioscience) for 30 minutes at 4 °C. The cells were then stained with the antibodies against the intracellular targets for 30 minutes at 4 °C. The cells were washed twice in FACS buffer and measured on the BD LSR Fortessa with FACSDiva software. Analysis was performed with FLowJo (V.10.5.3).

#### Cell trace violet proliferation assay

For the proliferation analysis, CD8^+^ T cells were resuspended in PBS (1 million/ml) and stained with Cell Trace Violet (CTV) (Thermo Fisher Scientific, 2.5 μM) for 30 minutes at room temperature. The staining was stopped by adding three times the volume of culture medium. The cells were then washed and resuspended in culture medium. The cells were activated with anti-CD3/CD28 Dynabeads and cytokine mix, in presence or absence of recombinant PCSK9, for four days. The fluorescence signal was measured by flow cytometry. The data was analyzed using the proliferation tool in FLowjo (V.10.5.3). The proliferation index, as the sum of the cells in all generations divided by the computed number of original parent cells present at the start of the experiment was plotted.

#### RNA-Seq data

Differential expression results, performed using the CLC Genomics Workbench 12 (Qiagen, Hilden, Germany) with default settings, were obtained from online Table S1 of Saleh et al.^12^ gProfiler2 v0.2.3 was used to perform the functional enrichment analysis of underexpressed genes (adjusted p-value < 0.05), using Benjamini-Hochberg multiple testing correction.^56^

### Quantification and statistical analysis

GraphPad Prism software version 9 (GraphPad Software Inc., CA, USA) was used for data visualization and statistical analyses. The statistical tests applied, along with the number and type of replicates (n), are specified in the figure legends. Data shown in the manuscript were normalized to the corresponding untreated control for presentation purposes, whereas all statistical analyses were performed on non-normalized data. A p value < 0.05 was considered statistically significant.

## References

[1] Galassi C., Chan T.A., Vitale I., Galluzzi L. (2024). The hallmarks of cancer immune evasion. Cancer Cell.

[2] Mahboobnia K., Pirro M., Marini E., Grignani F., Bezsonov E.E., Jamialahmadi T., Sahebkar A. (2021). PCSK9 and cancer: Rethinking the link. Biomed. Pharmacother..

[3] Oza P.P., Kashfi K. (2023). The evolving landscape of PCSK9 inhibition in cancer. Eur. J. Pharmacol..

[4] Liu X., Bao X., Hu M., Chang H., Jiao M., Cheng J., Xie L., Huang Q., Li F., Li C.Y. (2020). Inhibition of PCSK9 potentiates immune checkpoint therapy for cancer. Nature.

[5] Hsu C.-Y., Abdulrahim M.N., Mustafa M.A., Omar T.M., Balto F., Pineda I., Khudair T.T., Ubaid M., Ali M.S. (2024). The multifaceted role of PCSK9 in cancer pathogenesis, tumor immunity, and immunotherapy. Med. Oncol..

[6] Seidah N.G., Prat A. (2022). The Multifaceted Biology of PCSK9. Endocr. Rev..

[7] Yuan J., Cai T., Zheng X., Ren Y., Qi J., Lu X., Chen H., Lin H., Chen Z., Liu M. (2021). Potentiating CD8+ T cell antitumor activity by inhibiting PCSK9 to promote LDLR-mediated TCR recycling and signaling. Protein Cell.

[8] Bonacina F., Moregola A., Svecla M., Coe D., Uboldi P., Fraire S., Beretta S., Beretta G., Pellegatta F., Catapano A.L. (2022). The low-density lipoprotein receptor–mTORC1 axis coordinates CD8+ T cell activation. J. Cell Biol..

[9] Brummel K., Eerkens A.L., de Bruyn M., Nijman H.W. (2023). Tumour-infiltrating lymphocytes: from prognosis to treatment selection. Br. J. Cancer.

[10] Gooden M.J.M., de Bock G.H., Leffers N., Daemen T., Nijman H.W. (2011). The prognostic influence of tumour-infiltrating lymphocytes in cancer: a systematic review with meta-analysis. Br. J. Cancer.

[11] Tang Y., Chen Z., Zuo Q., Kang Y. (2024). Regulation of CD8+ T cells by lipid metabolism in cancer progression. Cell. Mol. Immunol..

[12] Saleh R., Sasidharan Nair V., Toor S.M., Taha R.Z., Murshed K., Al-Dhaheri M., Khawar M., Petkar M.A., Abu Nada M., Al-Ejeh F., Elkord E. (2020). Differential gene expression of tumor-infiltrating CD8 + T cells in advanced versus early-stage colorectal cancer and identification of a gene signature of poor prognosis. J. Immunother. Cancer.

[13] Cerami E., Gao J., Dogrusoz U., Gross B.E., Sumer S.O., Aksoy B.A., Jacobsen A., Byrne C.J., Heuer M.L., Larsson E. (2012). The cBio Cancer Genomics Portal: An open platform for exploring multidimensional cancer genomics data. Cancer Discov..

[14] de Bruijn I., Kundra R., Mastrogiacomo B., Ngoc Tran T., Sikina L., Mazor T., Li X., Ochoa A., Zhao G., Lai B., et al. Analysis and Visualization of Longitudinal Genomic and Clinical Data from the AACR Project GENIE Biopharma Collaborative in cBioPortal. 10.1158/0008-5472.CAN-23-0816/3362081/can-23-0816.pdf PMC10690089 37668528

[15] Gao J., Arman Aksoy B., Dogrusoz U., Dresdner G., Gross B., Sumer S.O., Sun Y., Jacobsen A., Sinha R., Larsson E., et al. Integrative Analysis of Complex Cancer Genomics and Clinical Profiles Using the CBioPortal InTRODUcTiOn EQUiPMenT InSTRUcTiOnS Querying Individual Cancer Studies Viewing and Interpreting the Results Performing Cross-Cancer Queries Viewing Cancer Study Summary Data Viewing Genomic Alterations in a Single Tumor: Patient View Programmatic Access NOTeS anD ReMaRKS Complementary Data Sources and Analysis Options Future Directions; 2013. p. 6. http://www.adobe.com/products/illustrator.html. 10.1126/scisignal.2004088 PMC4160307 23550210

[16] Aaltonen L.A., Abascal F., Abeshouse A., Aburatani H., Adams D.J., Agrawal N., Ahn K.S., Ahn S.M., Aikata H., Akbani R. (2020). Pan-cancer analysis of whole genomes. Nature.

[17] Zelcer N., Hong C., Boyadjian R., Tontonoz P. (2009). LXR Regulates Cholesterol Uptake Through Idol-Dependent Ubiquitination of the LDL Receptor. Science.

[18] van Loon N.M., Lindholm D., Zelcer N. (2019). The E3 ubiquitin ligase inducible degrader of the LDL receptor/myosin light chain interacting protein in health and disease. Curr. Opin. Lipidol..

[19] Zhang S.-Z., Zhu X.D., Feng L.H., Li X.L., Liu X.F., Sun H.C., Tang Z.Y. (2021). PCSK9 promotes tumor growth by inhibiting tumor cell apoptosis in hepatocellular carcinoma. Exp. Hematol. Oncol..

[20] Yang Q.-C., Wang S., Liu Y.T., Song A., Wu Z.Z., Wan S.C., Li H.M., Sun Z.J. (2023). Targeting PCSK9 reduces cancer cell stemness and enhances antitumor immunity in head and neck cancer. iScience.

[21] Wang L., Li S., Luo H., Lu Q., Yu S. (2022). PCSK9 promotes the progression and metastasis of colon cancer cells through regulation of EMT and PI3K/AKT signaling in tumor cells and phenotypic polarization of macrophages. J. Exp. Clin. Cancer Res..

[22] Mei W., Faraj Tabrizi S., Godina C., Lovisa A.F., Isaksson K., Jernström H., Tavazoie S.F. (2025). A commonly inherited human PCSK9 germline variant drives breast cancer metastasis via LRP1 receptor. Cell.

[23] Fang S., Yarmolinsky J., Gill D., Bull C.J., Perks C.M., Davey Smith G., Gaunt T.R., Richardson T.G., PRACTICAL Consortium (2023). Association between genetically proxied PCSK9 inhibition and prostate cancer risk: A Mendelian randomisation study. PLoS Med..

[24] Li C.-Y., Wang W.T., Ma S.H., Lo L.W., Wu C.Y., Chang W.C., Chen Y.J., Chen T.L. (2025). Association of proprotein convertase subtilisin/kexin type-9 inhibitors with risk of nonmelanoma skin cancer: a retrospective cohort study. Br. J. Dermatol..

[25] Shipkova M., Wieland E. (2012). Surface markers of lymphocyte activation and markers of cell proliferation. Clin. Chim. Acta.

[26] Chen Y., Yu D., Qian H., Shi Y., Tao Z. (2024). CD8+ T cell-based cancer immunotherapy. J. Transl. Med..

[27] Yan C., Zheng L., Jiang S., Yang H., Guo J., Jiang L.Y., Li T., Zhang H., Bai Y., Lou Y. (2023). Exhaustion-associated cholesterol deficiency dampens the cytotoxic arm of antitumor immunity. Cancer Cell.

[28] Flinsenberg T.W.H., Compeer E.B., Koning D., Klein M., Amelung F.J., van Baarle D., Boelens J.J., Boes M. (2012). Fcγ receptor antigen targeting potentiates cross-presentation by human blood and lymphoid tissue BDCA-3+ dendritic cells. Blood.

[29] Spel L., Nieuwenhuis J., Haarsma R., Stickel E., Bleijerveld O.B., Altelaar M., Boelens J.J., Brummelkamp T.R., Nierkens S., Boes M. (2018). Nedd4-Binding Protein 1 and TNFAIP3-Interacting Protein 1 Control MHC-1 Display in Neuroblastoma. Cancer Res..

[30] Hosken N.A., Bevan M.J. (1990). Defective Presentation of Endogenous Antigen by a Cell Line Expressing Class I Molecules. Science.

[31] Bossi G., Gerry A.B., Paston S.J., Sutton D.H., Hassan N.J., Jakobsen B.K. (2013). Examining the presentation of tumor-associated antigens on peptide-pulsed T2 cells. OncoImmunology.

[32] Tomlinson B., Hu M., Zhang Y., Chan P., Liu Z.-M. (2017). Alirocumab for the treatment of hypercholesterolemia. Expert Opin. Biol. Ther..

[33] Robinson J.G., Farnier M., Krempf M., Bergeron J., Luc G., Averna M., Stroes E.S., Langslet G., Raal F.J., El Shahawy M. (2025). Efficacy and safety of alirocumab in reducing lipids and cardiovascular events. N. Engl. J. Med..

[34] Fellin R., Arca M., Zuliani G., Calandra S., Bertolini S. (2015). The history of autosomal recessive hypercholesterolemia (ARH): From clinical observations to gene identification. Gene.

[35] Leigh T., Kawai T., Preston K., Kelemen S., Okune R., St Paul A., Corbett C., Peluzzo A.M., Yu J., Scalia R.G., Autieri M.V. (2022). Deletion of LDLRAP1 Induces Atherosclerotic Plaque Formation, Insulin Resistance, and Dysregulated Insulin Response in Adipose Tissue. Am. J. Pathol..

[36] Ma X., Bi E., Lu Y., Su P., Huang C., Liu L., Wang Q., Yang M., Kalady M.F., Qian J. (2019). Cholesterol Induces CD8+ T Cell Exhaustion in the Tumor Microenvironment. Cell Metab..

[37] Madison B.B. (2016). Srebp2: A master regulator of sterol and fatty acid synthesis1. J. Lipid Res..

[38] Wang F., Beck-García K., Zorzin C., Schamel W.W.A., Davis M.M. (2016). Inhibition of T cell receptor signaling by cholesterol sulfate, a naturally occurring derivative of membrane cholesterol. Nat. Immunol..

[39] Yin J., Fu J., Shao Y., Xu J., Li H., Chen C., Zhao Y., Zheng Z., Yu C., Zheng L., Wang B. (2023). CYP51-mediated cholesterol biosynthesis is required for the proliferation of CD4+ T cells in Sjogren’s syndrome. Clin. Exp. Med..

[40] Yang W., Bai Y., Xiong Y., Zhang J., Chen S., Zheng X., Meng X., Li L., Wang J., Xu C. (2016). Potentiating the antitumour response of CD8+ T cells by modulating cholesterol metabolism. Nature.

[41] Kabouridis P.S., Janzen J., Magee A.L., Ley S.C. (2000). Cholesterol depletion disrupts lipid rafts and modulates the activity of multiple signaling pathways in T lymphocytes. Eur. J. Immunol..

[42] Kidani Y., Elsaesser H., Hock M.B., Vergnes L., Williams K.J., Argus J.P., Marbois B.N., Komisopoulou E., Wilson E.B., Osborne T.F. (2013). Sterol regulatory element–binding proteins are essential for the metabolic programming of effector T cells and adaptive immunity. Nat. Immunol..

[43] Angela M., Endo Y., Asou H.K., Yamamoto T., Tumes D.J., Tokuyama H., Yokote K., Nakayama T. (2016). Fatty acid metabolic reprogramming via mTOR-mediated inductions of PPARγ directs early activation of T cells. Nat. Commun..

[44] Ungvari Z., Menyhart O., Lehoczki A., Fekete M., Bianchini G., Győrffy B. (2025). PCSK9 expression and cancer survival: a prognostic biomarker at the intersection of oncology and geroscience. GeroScience.

[45] Courtney A.H., Lo W.-L., Weiss A. (2018). TCR Signaling: Mechanisms of Initiation and Propagation. Trends Biochem. Sci..

[46] Johansen J., Ramanathan V., Beh C.T. (2012). Vesicle trafficking from a lipid perspective. Cell. Logist..

[47] Behr F.M., Chuwonpad A., Stark R., van Gisbergen K.P.J.M. (2018). Armed and Ready: Transcriptional Regulation of Tissue-Resident Memory CD8 T Cells. Front. Immunol..

[48] Kaizuka Y., Douglass A.D., Varma R., Dustin M.L., Vale R.D. (2007). Mechanisms for segregating T cell receptor and adhesion molecules during immunological synapse formation in Jurkat T cells. Proc. Natl. Acad. Sci. USA.

[49] Luo Y., Yuan L., Liu Z., Dong W., Huang L., Liao A., Xie Y., Liu R., Lan W., Cai Y., Zhu W. (2024). Inhibition of PCSK9 Protects against Cerebral Ischemia‒Reperfusion Injury via Attenuating Microcirculatory Dysfunction. Neurochem. Res..

[50] Ragusa R., Rocchiccioli S., Del Turco S., Morlando A., Basta G., Scholte A., Neglia D., Caselli C. (2025). PCSK9 and coronary atherosclerosis progression beyond LDL-cholesterol in coronary artery disease patients. Eur. J. Clin. Invest..

[51] Mulder J.W.C.M., Reijman M.D., Kusters D.M., Boersma E., Alnouri F., Blom D.J., Catapano A.L., Cuchel M., Dann E.J., Freiberger T. (2025). Homozygous familial hypercholesterolemia is a life-limiting condition. J. Am. Coll. Cardiol..

[52] Mulder J.W.C.M., Tromp T.R., Al-Khnifsawi M., Blom D.J., Chlebus K., Cuchel M., D'Erasmo L., Gallo A., Hovingh G.K., Kim N.T. (2024). Sex Differences in Diagnosis, Treatment, and Cardiovascular Outcomes in Homozygous Familial Hypercholesterolemia. JAMA Cardiol..

[53] Krogh H.W., Svendsen K., Igland J., Mundal L.J., Holven K.B., Bogsrud M.P., Leren T.P., Retterstøl K. (2019). Lower risk of smoking-related cancer in individuals with familial hypercholesterolemia compared with controls: a prospective matched cohort study. Sci. Rep..

[54] Xiao M., Xu J., Wang W., Zhang B., Liu J., Li J., Xu H., Zhao Y., Yu X., Shi S. (2023). Functional significance of cholesterol metabolism in cancer: from threat to treatment. Exp. Mol. Med..

[55] Gelissen I.C., Brown A.J. (2017).

[56] Kolberg L., Raudvere U., Kuzmin I., Vilo J., Peterson H. (2020). gprofiler2 – an R package for gene list functional enrichment analysis and namespace conversion toolset g:Profiler [version 2; peer review: 2 approved]. F1000Res.

