# Supplementary material for: PCSK9-mediated degradation of cell-surface LDL receptors impairs human CD8+ T cell effector functions
Source: iScience. 2026 Feb 7;29(3):114859. doi: 10.1016/j.isci.2026.114859 (PMC12936826; doi:10.1016/j.isci.2026.114859)
Supplement: Document S1. Figures S1–S4 and Table S1 [file mmc1.pdf]

iScience, Volume *29*

Supplemental information

PCSK9-mediated degradation of cell-surface LDL receptors impairs human CD8+ T cell effector functions

Angela Markovska, Lara F. Lommers, Alejandra Bodelón, Patrick Greve, Leonie C. van Vark-van der Zee, Monique T. Mulder, Jeanine E. Roeters van Lennep, Noam Zelcer, Henk S. Schipper, and Marianne Boes

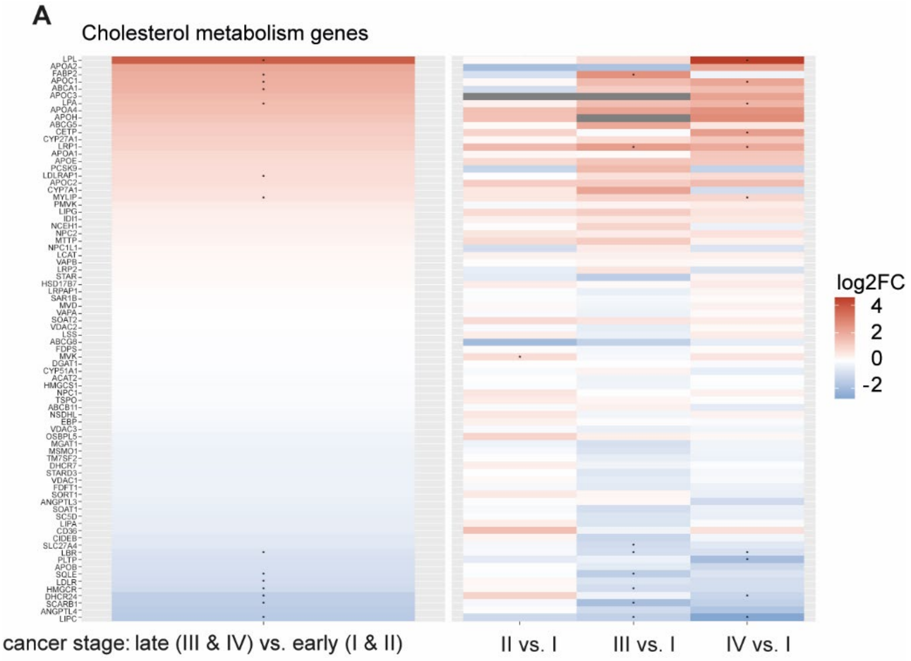

Figure S1. Tumor-infiltrating lymphocytes (TILs) from patients with colorectal cancer show reliance on cholesterol synthesis and uptake. (**A**) Heatmap of genes involved in the Cholesterol Metabolism KEGG (hsa04979) and Wiki Pathway (WP5304) in TILs from late-stage versus early-stage colorectal cancer patients. The left panel compares late-stage to early-stage colorectal cancer, while the right panel illustrates gene expression changes across disease progression (stage II vs. I, stage III vs. I, and stage IV vs. I). Significance is shown with '*' and values are shown as log_2foldchange (log_2FC).

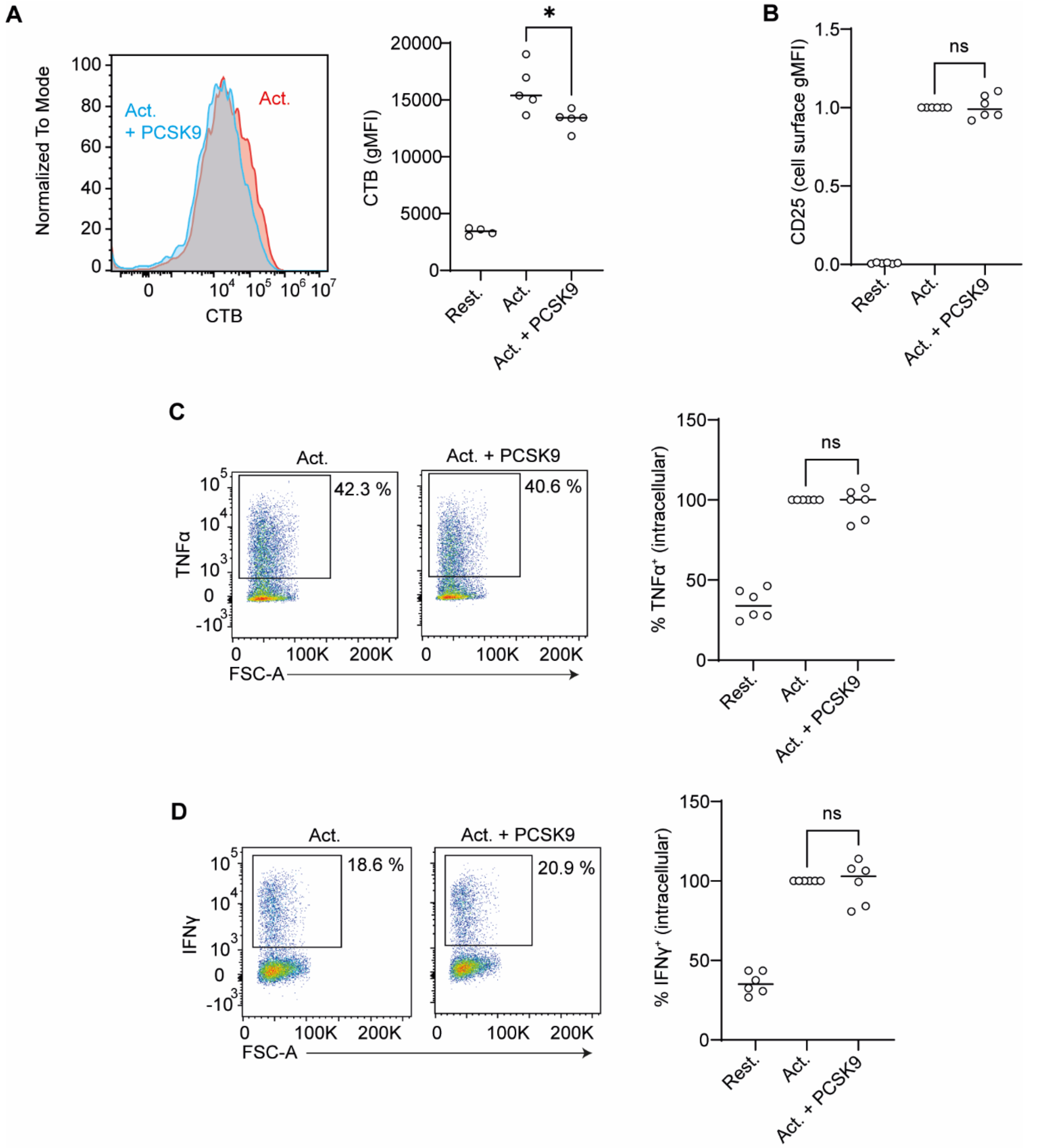

Figure S2. PCSK9-treated activated CD8+ T cells show decreased lipid microdomain formation, and no difference in CD25, TNFα, and IFNγ compared to controls. (**A**) gMFI of cholera toxin B (CTB)-FITC staining of CD8+ T cells. Two-tailed Mann-Whitney test where, n=5 healthy donors. Each dot represent data from a separate donor and lines depict medians. CD8+ T cells were activated with anti CD3/28 as shown in Fig. 2A (PCSK9 supplementation on Day 1 and 2, measurement on day 3). (**B**) Normalized CD25 cell surface geometric mean fluorescence intensity (gMFI) levels on CD8+ T cells measured at day 3 of activation with flow cytometry. Two-tailed Mann-Whitney test, n=6 healthy donors. Each dot represent data from a separate donor and lines depict medians. (**C, D**) Intracellular TNFα and IFNγ levels measured with flow cytometry. Two-tailed Mann-Whitney test where, n=6 healthy donors. Each dot represent data from a separate donor and lines depict medians.

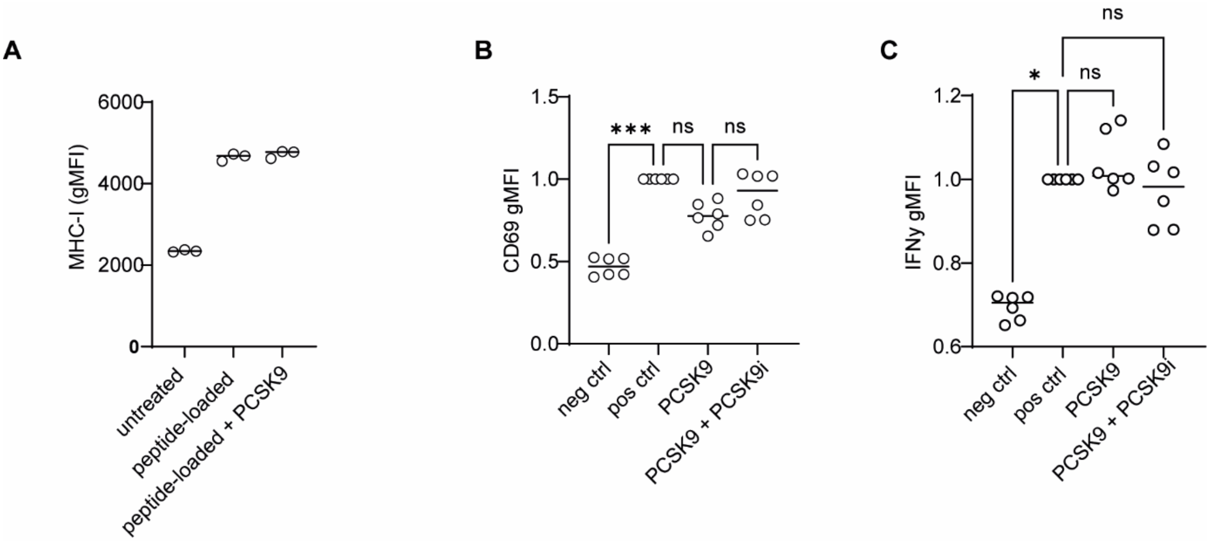

Figure S3. CD8+ T cells specific to NLVPMVATV/HLA-A2 complexes were co-cultured with T2 cells loaded with NLVPMVATV peptide or the irrelevant MART-1-derived ELAGIGILTV peptide. NLVPMVATV peptide is derived from the cytomegalovirus (CMV) protein pp65. In the figure, 'neg ctrl' represents the co-culture in presence of the irrelevant MART-1-derived ELAGIGILTV peptide, while in all other conditions CMV-derived NLVPMVATV peptide was added. Where indicated, recombinant PCSK9 (10 μg/ml) and Alirocumab (2 μM) were supplemented to the co-culture. (**A**) MHC-I cell surface levels on T2 cells measured with flow cytometry. (**B**) Normalized CD69 cell surface expression on the CMV-specific CD8+ T cells measured with flow cytometry. Kruskal-Wallis test with Dunn's multiple comparisons test, ***p<0.001, n=6 independent replicates. Each dot represents an independent replicate and lines depict medians. (**C**) Normalized intracellular IFNγ levels in the CMV-specific CD8+ T cells measured with flow cytometry. 3 hours before measuring cells were treated with GolgiStop (1500x, BD Biosciences). Kruskal-Wallis test with Dunn's multiple comparisons test, *p<0.05, n=6 independent replicates. Each dot represents an independent replicate and lines depict medians.

A

Mutation details	Classification
Compound heterozygous: Leiden-3 mutation (4.4 kb duplication in exon 12) and Capetown-2 mutation (2.5 kb deletion in exons 7 and 8) of *LDLR*	LDLR defective/null
Homozygous c.406C>T (p.Gln136Ter) mutation in *LDLRAP1*	ARH (autosomal recessive hypercholesterolemia)
Homozygous c.1685_1687del (p.His562del) in exon 11 of *LDLR*	LDLR null/null
Homozygous c.1054G>A (p.Gly352Asp) mutation in exon 8 and c.2417_2418 (p.Val806GlyfsTer11) mutation in exon 17 of *LDLR*	LDLR defective/null

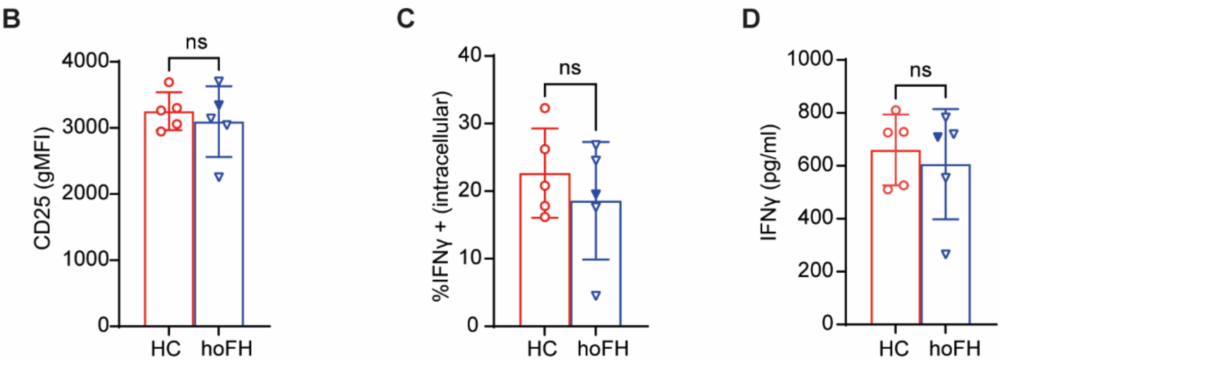

Figure S4: CD8+ T cells from homozygous familial hypercholesterolemia (hoFH) patients confirm importance of low-density lipoprotein receptor (LDLR) signaling for CD8+ T cell function. (**A**) A description of the mutations of patients with homozygous familial hypercholesterolemia (hoFH) included in our study. (**B**) Cell surface CD25 levels measured with flow cytometry, Two-tailed Mann-Whitney test, (n=5 HC and n=5 hoFH). Each dot represent data from a separate donor and lines depict medians. (**C**) Intracellular IFNγ levels measured with flow cytometry. Two-tailed Mann-Whitney test, (n=5 HC and n=5 hoFH). Each dot represent data from a separate donor and lines depict

medians. (**D**) Secreted IFNγ levels measured with ELISA. Two-tailed Mann-Whitney test, (n=5 HC and n=5 hoFH). Each dot represent data from a separate donor and lines depict medians.

Tabe S1. Primer sequences used for SYBR green qPCR.

Target gene	Forward primer sequence	Reverse primer sequence
HMGCR	TTCGGTGGCCTCTAGTGAGA	GATGGGAGGCCACAAAGAGG
LDLR	AGCTACCCCTCGAGACAGAT	ACTGTCCGAAGCCTGTTCTG
